# Energy expenditure and enjoyment of instructor-led exercise videos for children ages 7–13

**DOI:** 10.1371/journal.pone.0333283

**Published:** 2025-10-03

**Authors:** Mariam A. Habring, Penelope J. Friday, Anna Schwartz, Hala I. Abbas, Lexie R. Beemer, Rebecca E. Hasson

**Affiliations:** School of Kinesiology, University of Michigan, Ann Arbor, Michigan, United States of America; Neighborhood Physical Therapy, UNITED STATES OF AMERICA

## Abstract

**Background:**

The Interrupting Prolonged sitting with ACTivity (InPACT) at Home intervention provides youth with access to virtual, instructor-led exercise videos to promote physical activity in the home. While this program has had statewide reach, there is a need to understand the efficacy of the exercise videos to (1) induce positive cardiometabolic benefits in youth and (2) promote exercise enjoyment in the home.

**Purpose:**

The purpose of this study was to determine and compare the effects of the InPACT at Home exercise videos on physical activity energy expenditure and physical activity enjoyment in children ages 7–13 years.

**Methods:**

Thirty-nine participants ages 7–13 years were recruited to participate in this study. Participants completed five, 8-minute InPACT at Home exercise videos (cardio, strength, sports skills, and mindfulness) and one, 8-minute control video (sedentary). Physical activity energy expenditure was assessed using indirect calorimetry. Physical activity enjoyment was assessed using the Physical Activity Enjoyment Scale.

**Results:**

Exercise videos elicited significantly higher physical activity energy expenditure and enjoyment compared to the control video (p < 0.05). There were also significant differences in physical activity energy expenditure by video type with the highest energy expenditure recorded during the cardio videos (24.84 ± 9.14 kcals) followed by the strength (20.97 ± 9.97 kcals), sports skills (20.66 ± 7.72 kcals), and mindfulness videos (17.34 ± 7.80 kcals, p < 0.05). There were no significant differences in physical activity enjoyment by exercise video type (all p’s > 0.05).

**Conclusion:**

On average, children expended approximately 22 kcals while engaging in the InPACT at Home videos and rated all the videos as enjoyable. These findings highlight the potential of the InPACT at Home intervention to provide sustainable opportunities for youth to engage in health-enhancing physical activity in the home.

## Introduction

Developing and disseminating youth physical activity programs became a top public health priority for behavioral interventionists working to maintain child health, well-being, and achievement during the COVID-19 pandemic [1,2]. Physical activity is one of the most efficacious pathways to promote mental and physical health, prevent disease, and in the context of the pandemic, bolster a stronger immune system [3–5]. Yet, with school closures limiting opportunities to engage in physical education, recess, and extracurricular athletic activities [6,7], there was an urgent and unmet need to increase structured physical activity opportunities for children and adolescents in the home. Indeed, the lack of school- and community-based physical activity programs during the first year of the pandemic resulted in a 17-minute decline in children’s daily moderate-to-vigorous physical activity levels [8], along with a 9% increase in childhood obesity [9], and a widening of the preexisting academic achievement gap [10]. To address this issue, physical activity interventions must be designed to improve accessibility for individuals with limited access, a challenge that was further exacerbated during the pandemic.

The Interrupting Prolonged Sitting with Activity (InPACT) at Home intervention was developed during the pandemic and designed to meet the need for increasing youth physical activity opportunities in the home environment [11]. InPACT at Home is an evidence-informed physical activity intervention that utilizes high-quality, instructor-led exercise videos to promote activity for youth at home [12]. InPACT at Home was rapidly adapted from InPACT, a classroom-based physical activity intervention where the core element of providing 20 minutes of exercise throughout the day using instructor-led exercise videos was maintained [13]. Physical education teachers, fitness professionals, pediatric exercise physiologists, and athletes were hired to develop exercise videos that were developmentally appropriate and could be completed at home with no or minimal equipment. The intervention was disseminated via a program website (inpactathome.umich.edu) and public broadcasting television (https://www.michiganlearning.org/inpactathome/).

InPACT at Home exercise videos were specifically designed to promote cardiometabolic health – an essential target for reducing long-term risk of chronic diseases such as type 2 diabetes and cardiovascular disease [14] – by using a variety of intermittent exercises (video duration: 6–8 minutes; video type: cardio-based, strength-based, sports skills, and mindfulness) [11], commonly referred to as “exercise snacks” [15]. Improving cardiometabolic outcomes such as weight status, waist circumference, and physical activity energy expenditure (PAEE) in childhood can reduce disease risk into adulthood [14,16–18].

Previous research has demonstrated the utility of intermittent exercise to improve weight status and waist circumference in children and adolescents [19,20]. In a cohort of 2498 youth ages 8–17 years, Mark & Janssen determined that moderate-to-vigorous physical activity accumulated in doses of five or more minutes predicted adiposity status independent of the total volume of moderate-to-vigorous physical activity [19]. Willis et al. demonstrated that children who accumulated moderate-to-vigorous physical activity with a higher percentage of short bouts (5–10 minutes) had a lower BMI percentile and waist circumference compared to children who participated in longer duration physical activities [20]. These observational studies provide preliminary evidence for the efficacy of intermittent exercise performed at a moderate-to-vigorous intensity to prevent excess weight gain and enhance cardiometabolic outcomes in children and adolescents.

The InPACT at Home exercise videos were also designed to promote physical activity enjoyment – a key factor in motivating children to engage in and sustain physical activity over time [21]. How children feel during and after physical activity strongly influences their likelihood of engaging in it again [22–24]. Enjoyment is a well-established predictor of physical activity participation in both the short and long term [22,25–27], and may be especially important for sustaining activity in home settings where external reinforcement is limited. When children perceive physical activity as enjoyable, they are more likely to adhere to exercise routines, explore new types of movement, and develop lifelong healthy habits [28–31]. Previous research from our team has shown that intermittent exercise is enjoyable for children across weight statuses, and among those with chronic conditions like asthma, suggesting broad applicability and appeal [32,33].

Although the InPACT at Home intervention has achieved substantial reach (estimated 15,000–20,000 daily broadcast viewers statewide) [11] little is known about its short-term effects on PAEE and physical activity enjoyment—two important indicators of whether the intervention can produce the metabolic and motivational responses needed to support long-term cardiometabolic health. The purpose of this study was to determine and compare the acute effects of the InPACT at Home exercise videos on PAEE and enjoyment in children ages 7–13 years. We hypothesized that the videos would elicit significantly higher levels of PAEE compared to sedentary control videos and be rated as enjoyable by participants, supporting their potential as a scalable and sustainable tool to promote health-enhancing physical activity beyond the pandemic.

## Methods

### Participants

Children between the ages of 7–13 years were recruited from the greater Ann Arbor and Ypsilanti, Michigan areas to participate in a laboratory-based study. Participants were excluded if: (1) they were taking medications or diagnosed with diseases that could influence appetite, exercise ability, body composition, mood, or glucose metabolism; or (2) they were previously diagnosed with any major illness/health condition since birth; (3) were diagnosed with clinical depression or any other mental health disorder that may have influenced mood or emotions; (4) answered “yes” to one or more questions on the Physical Activity Readiness Questionnaire (PAR-Q) [34] indicating the participant was unable to fully participate in exercise without inducing harm; and (5) unable to complete questionnaires and physical tasks. After confirming eligibility, participants and their parents were provided with a full description of the study and signed an informed assent and consent document, respectively. The University of Michigan institutional review board approved this study (HUM00211507).

### Study design

**Fig 1:**
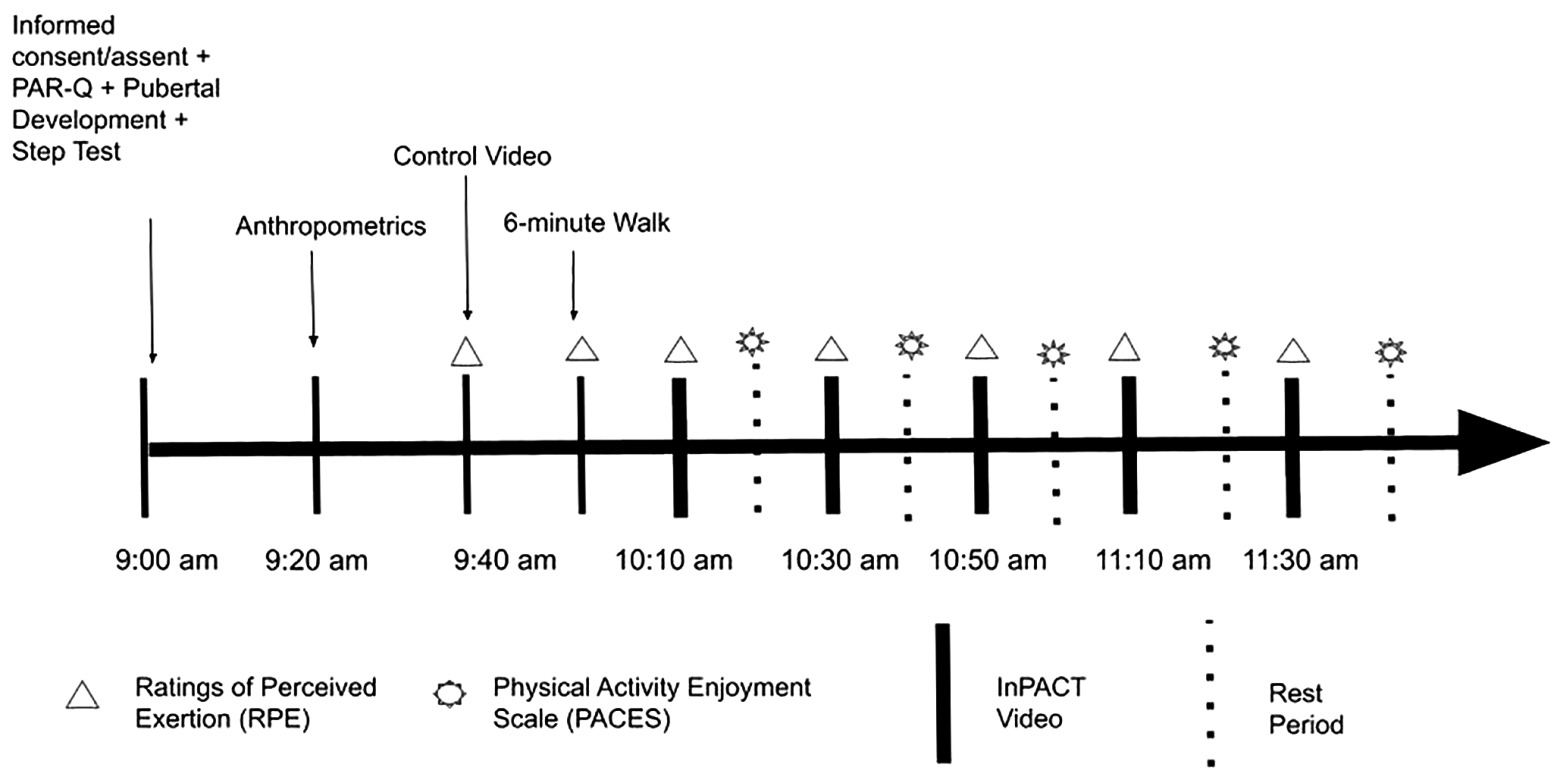
Study timeline.

The timeline for the experimental condition day is presented in Fig 1. Participants were instructed to arrive at the Childhood Disparities Research Laboratory between the hours of 0800 and 1100 wearing gym shoes and athletic clothing. During the experimental condition day, participants completed anthropometric measurements and demographic questionnaires, a fitness test, a control video, a warm-up walk, and five exercise videos. PAEE was measured throughout the experimental condition day, specifically during activity periods (i.e., warm-up walk, exercise videos, etc.). Measurements are described below in the order in which they were performed. The duration of the experimental condition day was approximately 3–4 hours.

### Pretesting measures

#### Anthropometric measurements and demographic questionnaires.

Height and body weight were measured to the nearest 0.1 cm and nearest 0.1 kg, respectively, using a ShortBoard® stadiometer (Weigh and Measure, LLC, Olney, MD) and an electronic scale (InBody, Cerritos, CA) using standardized procedures developed by Irwin Shorr [35]. Body mass index (BMI) was calculated with measured height and weight, as well as the participant’s sex and exact age on the date of data collection. BMI percentiles and corresponding weight status classifications were determined using the Centers for Disease Control and Prevention (CDC) BMI-for-age calculator, which applies standard percentile cut-offs: underweight (<5th percentile), healthy weight (5th–84th), overweight (85th–94th), obese (≥95th percentile), and severely obese (≥99 percentile) [36]. Information collected regarding sex, age, and race and ethnicity were based on self-report and parent report.

#### Cardiorespiratory fitness.

Participants’ cardiorespiratory fitness was assessed using the standardized Kasch Pulse Recovery (KPR) three-minute step test [37]. This test involved participants facing a 12-inch step and stepping on and off to the beat of a metronome. The metronome went at a pace of 96 beats per minute. Participants followed a cadence of first leg up, second leg up, first leg down, and second leg down. One minute post-test, heart rate was collected using the Polar Beat app and Polar H10 heart rate monitor (Polar Electro Inc., Lake Success, NY). Fitness level was then classified on a scale from very poor to excellent according to reference values for post-exercise heart rate according to age and sex [37]. After completing the fitness test, participants engaged in approximately a 10-minute rest period prior to the beginning of the experimental condition.

### Experimental condition

#### Control video.

Prior to completing the exercise videos, a neutral-affect educational video was shown to collect a baseline assessment of PAEE watching a video without engaging in physical activity. During the video viewing, participants were instructed to sit quietly throughout the duration of the video (approximately 8 minutes).

#### Warm-up activity.

On completion of the control video, participants were given a self-selected 5–10-minute rest break followed by a 6-minute self-paced walk as their warm-up. Participants were given another 5–10-minute break after the self-paced walk.

#### Exercise videos.

Following the warm-up activity, participants completed five, 8-minute InPACT at Home videos (40 minutes of total activity). There were four different types of videos from which the five videos were randomly selected, including (1) cardio, which involved high-intensity exercises and circuit training, (2) strength, which involved low-impact weight training and plyometrics, (3) sports skills, which involved agility and activities drawn from specific sports such as gymnastics and basketball, and (4) mindfulness, which involved yoga, meditation, and balance training. Participants were told to follow along with the video to the best of their ability; researchers provided equipment to be used, if applicable. For example, water bottles were provided to standardize the protocol for the videos which used weights. If a partner activity was shown in a video, the researcher acted as the participant’s partner to complete the exercise. Researchers did not provide instructions on completing any exercises nor offer encouragement throughout the videos. Participants were given a self-selected 5–10-minute break in between each video. Participants were offered a standardized snack of 100–200 kcals after the second video. An additional five minutes of rest was given to eat the snack as necessary.

#### Randomization.

A subset of 91 InPACT at Home videos was tested that did not include exercises performed while the participant was laying on their back (i.e., abdominal crunches) due to PAEE equipment limitations. Each participant completed five randomly selected InPACT at Home videos, which could include any combination and order of cardio, strength, sports skills, and mindfulness videos. The randomization procedure was designed to allow for each video to be completed by approximately three unique participants.

### Outcome measures

#### PAEE.

Participants’ PAEE was assessed using the COSMED K5 portable metabolic system (Oxycon Mobile, Cardinal Health, Dublin, Ohio), which provides breath-by-breath analysis. Calibration procedures—including reference gas (16% O₂, 5% CO₂), flowmeter, and scrubber checks—were completed and verified before each session using manufacturer-recommended protocols. Participants wore Hans Rudolph masks (XS–M) and the K5 unit secured in its standard backpack to allow freedom of movement. The device recorded continuous data during each activity session, including the control video, warm-up walk, and five exercise videos, with testing periods aligned to activity start and stop times.

PAEE data were processed using OMNIA software and averaged in 30-second epochs to capture cumulative energy expenditure. Washout periods of 5–10 minutes occurred between videos. The COSMED K5 has been validated for measuring total PAEE in both children and adults and is considered a criterion measure for energy expenditure [38,39].

#### Physical activity enjoyment.

The revised Physical Activity Enjoyment Scale [23] was used to assess enjoyment of physical activity completed immediately after each individual exercise video. Participants were asked to reflect on how they felt about the physical activity. Example questions included: “When I was physically active, I enjoyed it; when I was physically active, I felt bored; when I was physically active, I found it pleasurable.” The scale is a combination of 16 positive and negative statements. The responses were scored on a 5-point Likert scale [1 (disagree a lot) to 5 (agree a lot)]. Of the 16 statements, seven statements were reverse scored. An enjoyment score was calculated by averaging the scores. A higher total average corresponds to higher levels of enjoyment. The revised Physical Activity Enjoyment Scale has been validated in children, Cronbach’s α = .89 [40].

### Statistical analysis

All analyses were conducted in SPSS (version 27.0; IBM Corp, Armonk, NY). Results are expressed as means and standard error. Changes in PAEE and enjoyment were analyzed using a two-way repeated measures analysis of variance (ANOVA), where the main effects of condition (control, exercise), sex, age, and type of exercise (mindfulness, strength, sports skills, cardio) were examined. If Mauchley’s test of sphericity was violated, data were corrected using Greenhouse Geisser epsilon (ε). When significant differences across experimental conditions and time were identified, post hoc pairwise comparisons with Bonferroni adjustments were conducted. An alpha level of p < 0.05 was used for all analyses.

## Results

### Participant characteristics

A total of 41 individuals were recruited and deemed eligible to participate in the study. Two Caucasian/white participants (one female, one male) withdrew before completion of the study due to lack of desire to continue the study. The remaining 39 participants completed five, 8-minute exercise videos and all study questionnaires. A two-tailed post hoc power analysis was performed using G*Power (Version 3.1.9.7; Heinrich-Heine-Universität Düsseldorf, Germany). With a sample size of 39 participants, the analysis had 80% power to detect a medium effect size (d = 0.50) in the mean differences of matched pairs for a dependent variable.

Participant characteristics are presented in Table 1. A total of 21 females (54%) and 18 males aged 7–13 years completed the study protocol with 15% of the sample being non-white, and 20% Hispanic. According to CDC classifications for weight status, among 41 children, seven were classified as overweight, three as obese, three as severely obese, and two underweight. The remaining 13 children were of healthy weight. On average, participants were classified as having good fitness levels, which is consistent with nationally representative samples of children in this age range [41].

**Table 1:** Participant demographics and averages.

Sex, female, %	54
Race, nonwhite, %	20
Hispanic, %	17
Age, y	9.86 ± 2.00
Height, cm	140.48 ± 14.98
Weight, kg	38.30 ± 16.15
BMI, kg/m^2, % healthy weight	63.41
BMI, kg/m^2, % obese or overweight	24.39
Fitness Level, % poor	7.32
Fitness Level, % sufficient	14.63
Fitness Level, % good	17.07
Fitness Level, % very good	24.39
Fitness Level, % excellent	36.58

### PAEE

There were significant differences in overall PAEE between the sedentary control video, and exercise videos (p < 0.0001) with participants expending significantly more kcals during the exercise videos (22.00 ± 9.25 kcals) compared to the control video (9.64 ± 2.25 kcals). On average, children burned 22 kcals during each InPACT video, resulting in a total energy expenditure of approximately 110 kcals after completing all five videos.

**Fig 2:**
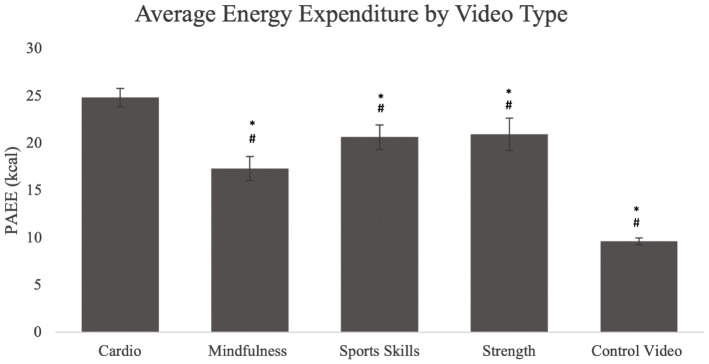
Average physical activity energy expenditure (PAEE) by video type. Hashtags (#) show significant differences between respective video categories and control video, while asterisks (*) show significant differences between respective video categories compared to the cardio videos.

The average PAEE by exercise video type is shown in Fig 2. All InPACT at Home video types resulted in significantly higher PAEE compared to the control video (p < 0.0001). There were also significant differences between the cardio and mindfulness videos (24.84 ± 9.14 kcal vs 17.34 ± 7.80 kcal, d = −0.862, p < 0.0001), cardio and strength videos (24.84 ± 9.14 kcal vs 20.97 ± 9.97 kcal, d = −0.579, p < 0.005), and cardio and sports skills videos (24.84 ± 9.14 kcal vs 20.66 ± 7.72 kcal, d = −0.503, p < 0.05). There were no significant differences in PAEE when comparing other video types to each other (p > 0.05).

**Fig 3:**
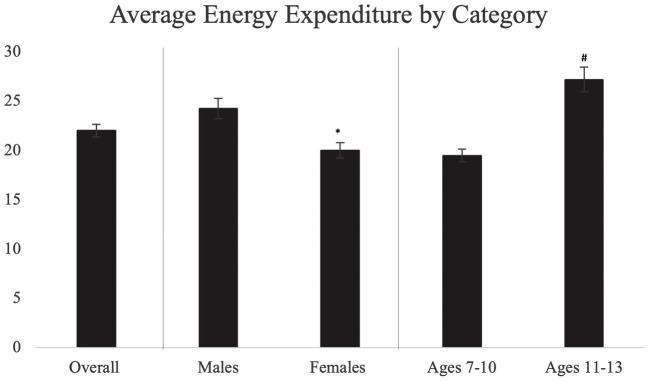
Average physical activity energy expenditure (PAEE) overall, by biological sex, and age. Hashtags (#) show significant differences between respective age groups, while asterisks (*) show significant differences between biological sex.

The average PAEE overall, by gender and by age, is shown in Fig 3. There were significant differences in PAEE between males and females (p < 0.005) with males expending significantly more kcals during exercise compared to females (24.23 ± 10.04 kcal vs 19.99 ± 7.93 kcals, d = 0.400). There were significant differences in PAEE by age group (p < 0.005), with children ages 11–13 years old expending more kcals during exercise compared to children ages 7–10 years old (27.18 ± 10.10 vs 19.48 ± 7.60, d = 0.166).

### Physical activity enjoyment

**Fig 4:**
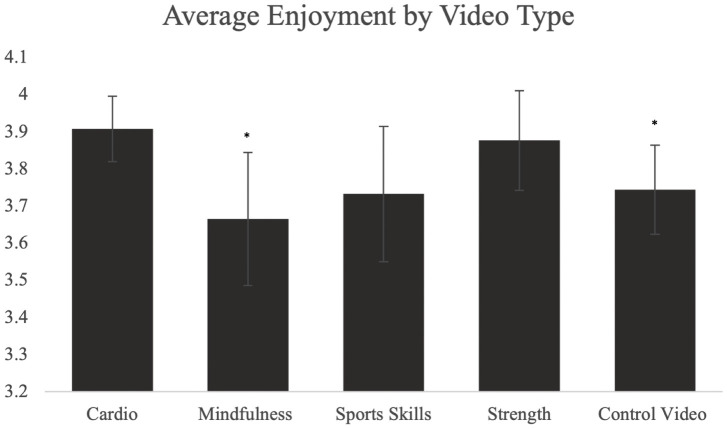
Average enjoyment based on video type. Asterisks (*) show significant differences between respective video categories compared to the cardio videos.

There were no significant differences in enjoyment between the control video and the exercise videos (3.74 ± 0.76 vs 3.80 ± 1.05, p > 0.05). The average PACES scores based on video type are presented in Fig 4. There were significant differences in physical activity enjoyment between the cardio and control videos (p < 0.05), with the cardio video eliciting greater enjoyment (3.91 ± 0.84 vs 3.74 ± 0.76, d = 0.319). There was a significant difference in enjoyment between the cardio and mindfulness videos (p < 0.05), with the cardio video eliciting greater enjoyment compared to the mindfulness video (3.91 ± 0.84 vs 3.66 ± 1.12, d = −0.449). There were no significant differences in enjoyment when comparing other video types to each other (all p’s > 0.05).

**Fig 5:**
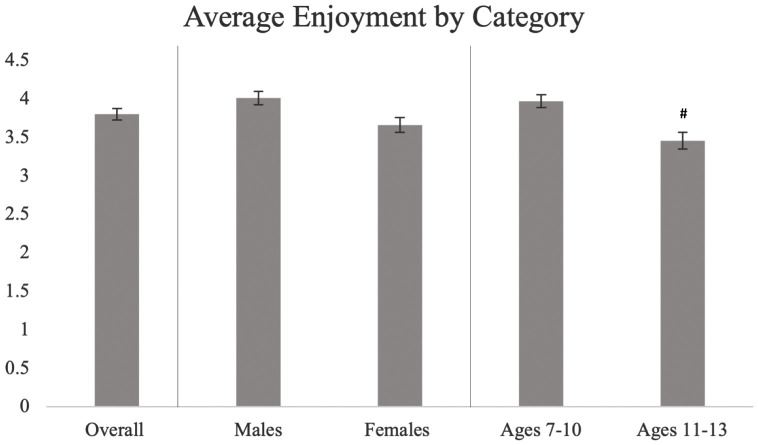
Average enjoyment overall, by biological sex, and age. Hashtags (#) show significant differences between respective age groups, while asterisks (*) show significant differences between biological sex.

The average PACES scores based on sex and age are shown in Fig 5. There was no significant difference in enjoyment between males and females (4.01 ± 0.84 vs 3.65 ± 0.99, p > 0.05). There was a significant difference in enjoyment by age (p < 0.05), with children ages 11−13 years old reporting lower enjoyment during exercise compared to children ages 7−10 years old (3.45 ± 0.87 vs 3.97 ± 0.97, d = −0.159).

## Discussion

The InPACT at Home program, which reached an estimated 15,000–20,000 daily viewers statewide, was created during the pandemic to provide universal access to health-enhancing physical activity when structured opportunities were limited [11]. Even after the pandemic, the program continues to help children and families incorporate more physical activity into their daily lives at home through public broadcasting on the Michigan Learning Channel. Despite the program’s large reach, it was important to evaluate the efficacy of these exercise videos in two areas: (1) their ability to induce positive cardiometabolic benefits in youth through increased PAEE, and (2) their role in promoting enjoyment of exercise at home.

On average, children burned 22 kcals during each InPACT video, resulting in a total energy expenditure of approximately 110 kcals after completing all five videos. Additionally, we expanded our analysis to examine age and sex differences in PAEE. As expected, older children (ages 11–13) and males expended significantly more calories, likely due to differences in body size, muscle mass, and exercise intensity [42]. These results indicate that, although the videos are generally effective, individual factors such as age and sex may influence the amount—or dose—of physical activity each child receives. The videos were also rated as enjoyable by all children, especially younger ones (ages 7–10), with no differences in enjoyment regarding sex. These findings suggest that InPACT at Home videos may effectively increase PAEE and enjoyment among children and encourage future participation in physical activity. However, the program may need to be tailored to ensure that all participants achieve a level of activity sufficient to support health benefits.

### PAEE: Benefits for youth

Consistent with other youth physical activity programs implemented in real-world settings, children in the present study expended an average of 22 kcals during the InPACT at Home videos (approximately 2.75–3.67 kcal/min). For example, the Take10! classroom physical activity program, which integrated physical activity into the instructional curriculum, reported that elementary-aged children expended an average of 25–37 kcals during their 10–11-minute lessons [43]. Another intervention aimed at reducing weight gain in elementary-aged children found that two, 10-minute physical activity sessions per day, which were led by classroom teachers, resulted in approximately 3.1 kcal expended per minute, or about 30 kcals per session [44]. An exergaming study looked at the energy expenditure of children (healthy weight and overweight) while doing three, 10-minute exergames, and found that children at a healthy weight expended about 3.51 kcal/min and children with overweight expended 4.75 kcal/min, with children overall expending around 35.1–47.5 kcal per exergame [45]. In contrast to these findings, we did not observe differences in PAEE by weight status when conducting post-hoc analyses in the present study. Nevertheless, these prior studies and present findings suggest that these short bursts of activity may effectively increase PAEE, which supports cardiometabolic health, and appear beneficial for children across weight statuses. Further, it is well established that intermittent physical activity can increase total daily energy expenditure without triggering compensatory changes in physical activity or caloric intake. Our previous research confirmed that children engaging in short activity bouts did not reduce other activity levels or increase food consumption in response [46,47]. As a result, regularly completing these videos may support pediatric weight management efforts by increasing 24-hour energy expenditure in a sustainable way. Regardless, further research is needed to investigate the long-term cardiometabolic benefits of the InPACT at Home program and potential public health impact. While we observed a statistically significant difference in energy expenditure between cardio and mindfulness videos (~7 kcal), the clinical relevance of this difference may seem modest. However, small, sustained increases in daily energy expenditure—on the order of 100 kcal/day—have been shown to help prevent gradual weight gain in children [48]. For example, engaging in five InPACT videos per day could result in an approximate additional expenditure of 110 kcals, which, over weeks and months, may support healthy weight maintenance or prevention of excess weight gain. This is especially relevant in the context of widespread decreases in physical activity observed during the COVID-19 pandemic [49]. Over time, consistently selecting higher-intensity cardio videos could contribute to meaningful changes; for example, an extra 7 kcal per video adds up to nearly 200 kcal per month, even if a cardio video is only selected once a day. Moreover, regular participation in intermittent moderate-to-vigorous physical activity, even in small doses, has been linked to improved cardiometabolic health markers such as insulin sensitivity and lipid profiles in youth, offering health benefits beyond calories burned [50]. It is important to note, however, that even with lower energy expenditure, the mindfulness videos provide complementary benefits. They support emotional regulation, calmness, and flexibility, and serve as an accessible entry point for children who may be less motivated or physically inclined to engage in vigorous activity [51–53]. Rather than diminishing the effectiveness of the overall program, the inclusion of mindfulness sessions may enhance engagement and sustainability by offering children greater choice and autonomy—key predictors of long-term adherence [54]. Thus, while cardio videos may yield greater immediate energy expenditure, mindfulness sessions may still play an important role in promoting holistic, sustainable physical activity behaviors over time.

### Enjoyment of exercise

Enjoyment is an important motivator in children’s physical activity participation as previous research has shown that enjoyment is a large predictor of whether one will continue being physically active [55]. The National Youth Physical Activity and Nutrition Survey analyzed the association between physical activity and activity type (such as sports, or individual activities) to assess whether enjoyment mediated the relationship between engagement and type of activity. The study found that providing a variety of physical activity options can help adolescents discover the activities they enjoy most and are more likely to continue doing. A study of elementary-aged children further found a significant correlation between physical activity and enjoyment. Children who reported greater enjoyment were more likely to engage in higher levels of physical activity, both in and out of school [56]. These findings confirm the importance of increasing physical activity enjoyment to better sustain participation among adolescents.

### Differences in enjoyment by age and sex

In the present study, children reported moderate-to-high levels of enjoyment after participating in the exercise videos, with no differences by sex. This aligns with prior research suggesting that boys and girls during middle childhood show similar enjoyment of structured physical activity, possibly due to shared preferences or broadly appealing video design [57]. However, enjoyment varied by age, with younger children (7–10 years) reporting greater enjoyment than older children (11–13 years).

This age difference may reflect younger children’s natural physical activity patterns, which are typically brief and intermittent. Prior studies show that fewer than 5% of children’s activity bouts last more than 10 minutes [58,59], with most lasting under 15 seconds [60]. Programs for younger children may therefore be more effective when incorporating short, frequent bursts of movement.

Developmental factors may also explain why older children reported lower enjoyment. During early adolescence, youth develop greater cognitive sophistication, heightened self-awareness, and shifting social priorities [61–63]. These changes may make them more critical of brief, guided videos and less intrinsically motivated to participate. Autonomy may also play a role- research consistently shows that adolescents enjoy physical activity more when they can choose the type, intensity, and format, which supports long-term adherence. Limited autonomy may therefore have contributed to lower enjoyment among older children compared to their younger peers [64–66].

Tailoring interventions to early adolescents by offering more choice, self-direction, and age-relevant content could enhance enjoyment. Evidence from the SPARK program, which increased physical activity and enjoyment among middle school students (mean age = 12.06 years) through student-centered, multi-activity PE lessons, supports this approach [67]. By fostering enjoyment-especially through autonomy-supportive and engaging content- programs like InPACT at home may improve adherence and sustain physical activity behaviors over time [68]. Future research should explore additional factors that contribute to the age-enjoyment relationship.

### Unique features of the InPACT at Home program

In contrast to many home-based physical activity interventions that require specialized equipment [69], paid subscriptions [70], or high levels of caregiver involvement [71], InPACT at Home is designed to be highly accessible. The brief, media-based exercise videos are freely available via public television and online platforms, require minimal space or equipment, and can be completed independently or with limited support. This approach enhances accessibility for families across diverse socioeconomic backgrounds and reduces common barriers to participation [72–74]. The use of certified physical education instructors, simple production formats, and flexible delivery methods distinguishes InPACT at Home from other home interventions and supports broad reach. At the same time, while these features promote usability, tailoring content to better reflect the preferences and autonomy needs of older children may be necessary to maximize engagement in this age group.

The InPACT at Home videos are brief, 6–8-minute sessions designed to reflect children’s natural movement patterns. Unlike continuous exercise, intermittent activities fit seamlessly into children’s daily routines, allowing them to incorporate movement without feeling overwhelmed or fatigued [75]. This method helps break up sedentary behavior and promotes physical activity throughout the day [13]. Children also find intermittent activity more enjoyable and less tiring, which fosters a positive experience and encourages future engagement in physical activity [32]. The InPACT at Home exercise videos effectively leverage this approach, providing a convenient, engaging, and enjoyable way for children to stay active and support their overall health and well-being.

### Strengths and limitations

Several limitations of the study should be considered. First, due to the limited sample size, the post hoc power analysis may not have provided sufficient power to detect subgroup differences by age, sex, or weight status, and the study was not powered to detect interaction effects. Second, although the study was conducted in a controlled laboratory environment to ensure standardization, this setting may not fully reflect how children engage with the InPACT at Home program in real-world home environments, where distractions, variability in adherence, and other contextual factors may influence participation and PAEE. Third, participants may not have followed the videos precisely as intended. While this reflects real-world usage patterns, it could affect the accuracy of PAEE estimates. To enhance ecological validity in future research, we recommend the use of wearable activity monitors (e.g., accelerometers or FitBits) to assess physical activity patterns and engagement with the program in home settings. This approach would enable more accurate tracking of adherence and energy expenditure in naturalistic environments and provide a stronger foundation for evaluating the program’s real-world effectiveness. Finally, although the sample included children with varying weight statuses, it lacked racial and ethnic diversity (85% White, 80% non-Hispanic), which limits the generalizability of our findings to more diverse populations. This is an important limitation, particularly for equity-focused interventions. While InPACT at Home was designed to reduce barriers to participation through universal free access, additional research is needed to examine its effectiveness and acceptability among racially and ethnically diverse groups. Ensuring that the program reflects the preferences and lived experiences of historically marginalized communities is essential for advancing equity in physical activity promotion.

Several strengths of the study should also be noted. First, the use of an objective, validated measure allowed for precise quantification of PAEE during video engagement. Second, the within-subjects design, in which each participant served as their own control, enabled direct comparisons of PAEE and enjoyment across conditions. Third, the inclusion of children with a range of weight statuses supports the generalizability of the videos for children with diverse anthropometric characteristics. Together, these strengths enhance the internal validity of the findings and provide a solid foundation for future studies evaluating the effectiveness of InPACT at Home in real-world settings.

## Conclusions

International reports indicate that while some children’s physical activity levels have improved post-pandemic, a “new normal” has emerged [76]. For example, in the United Kingdom, children are now more reliant on structured activities, such as sports, than on spontaneous play, which may create additional barriers for girls and children from lower socioeconomic backgrounds [76]. Although access to school-based programs has returned, home-based options like InPACT at Home can complement these efforts and help more children meet physical activity guidelines. This study found that the InPACT at Home exercise videos were both enjoyable and effective at inducing PAEE compared to a sedentary control condition. These brief, intermittent activity sessions align with children’s natural movement patterns and offer a developmentally appropriate, engaging strategy to promote daily physical activity. While the program was developed during the pandemic to promote equitable access, its continued availability through public broadcasting can support families in maintaining healthy movement routines at home.

Once the effectiveness is confirmed in real-world home settings, InPACT at Home may serve as a valuable tool for educators to reinforce classroom activity breaks, for parents to provide free, flexible movement options, and for public health professionals seeking scalable approaches to reduce sedentary time and address disparities in physical activity access. Future research should examine how tailoring content by age and incorporating autonomy-supportive features may enhance engagement, especially among older youth.

## Supporting information

S1 Data Table containing data for energy expenditure and enjoyment. Table A: Energy expenditure and enjoyment data. Table B: Baseline data.
(XLSX)
